# Decreasing prevalence of dementia in 85-year olds examined 22 years apart: the influence of education and stroke

**DOI:** 10.1038/s41598-017-05022-8

**Published:** 2017-07-21

**Authors:** Ingmar Skoog, Anne Börjesson-Hanson, Silke Kern, Lena Johansson, Hanna Falk, Robert Sigström, Svante Östling

**Affiliations:** Institute of Neuroscience and Physiology, Unite of Neuropsychiatric Epidemiology, Sahlgrenska Academy at the University of Gothenburg, Gothenburg, Sweden

## Abstract

Individuals aged 80 years and older constitute the fastest growing segment of the population worldwide, leading to an expected increase in dementia cases. Education level and treatment of vascular risk factors has increased during the last decades. We examined whether this has influenced the prevalence of dementia according to DSM-III-R using population-based samples of 85-year-olds (N = 1065) examined with identical methods 1986–87 and 2008–10. The prevalence of dementia was 29.8% in 1986–87 and 21.7% in 2008–10 (OR 0.66; 95%-CI: 0.50–0.86). The decline was mainly observed for vascular dementia. The proportion with more than basic education (25.2% and 57.7%), and the prevalence of stroke (20% and 30%) increased, but the odds ratio for dementia with stroke decreased from 4.3 to 1.8 (interaction stroke*birth cohort; p = 0.008). In a logistic regression, education (OR 0.70; 95%-CI 0.51–0.96), stroke (OR 3.78; 95%-CI 2.28–6.29), interaction stroke*birth cohort (OR 0.50; 95%-CI 0.26–0.97), but not birth cohort (OR 0.98; 95%-CI 0.68–1.41), were related to prevalence of dementia. Thus, the decline in dementia prevalence was mainly explained by higher education and lower odds for dementia with stroke in later born birth cohorts. The findings may be related to an increased cognitive reserve and better treatment of stroke in later-born cohorts.

## Introduction

Most dementias occur after age 80^1^, an age group estimated to increase from 120 to 391 million worldwide from 2012 to 2050^2^, leading to an increase in the number of people with dementia from 36 million in 2010 to 115 million in 2050^1^. The already enormous costs for dementia^3, 4^, and the suffering of those afflicted and their relatives, will thus increase in the coming decades if the age-specific prevalence remains constant. It was recently reported that the incidence of dementia has decreased during the last four decades^5–7^. However, prevalence is the best measure of disease burden^8^. If the age-specific prevalence changes, especially after age 80 years, it may influence the future numbers of individuals with dementia. Several studies indicate that the prevalence is decreasing^9^, but data are inconclusive due to too short time-intervals between examinations in several studies^10^, and regarding types of dementia as well as possible explanations for time-trends, such as the effect of increasing educational levels in later-born cohorts^8^.

The frequency of several risk and protective factors for dementia have changed during the last decades, and later-born birth cohorts have experienced different life-courses than earlier-born cohorts^8^. The incidence of stroke^11, 12^, levels of systolic blood pressure^13, 14^ and total cholesterol^14, 15^ is decreasing, while the prevalence of overweight and diabetes mellitus^16, 17^, as well as survival after stroke^11, 12^, increases. Educational achievement has increased during the last century, and later born cohorts perform better on cognitive tests than earlier born cohorts^18^. This may affect the cognitive reserve, which moderates the association between brain pathology and clinical outcome^19^. It has been suggested that individuals with better cognitive reserve develop dementia later, but decline faster when symptoms appear^19, 20^, and may exhibit a shorter phase of preclinical and mild dementia^21, 22^.

To study time-trends in dementia occurrence is challenging^8, 10^. Examinations and diagnostic methods need to be identical over time, preferably using algorithms and structured interviews to minimize shifting in clinical evaluations and diagnostic boundaries^8, 10^. Studies need to be population-based with personal examinations, as register data are influenced by changes in awareness of dementia among health professionals. Response rates needs to be similar over time to avoid differential response bias. Few studies have so far been able to meet these criteria. In 1986–87, we conducted a study on 85-year-olds in Gothenburg, Sweden^23^. The prevalence of dementia was 30%, and a surprisingly large proportion had vascular dementia or mixed dementia. It was concluded that vascular dementia might be more amenable to prevention and treatment than Alzheimer’s disease^23^.

The aim of this study was to estimate if the prevalence of dementia and its causes, and the impact of cerebrovascular disease and education on dementia prevalence, had changed in representative samples of 85-year-olds examined 1986–87 and 2008–10.

## Results

### Demographic factors

Demographic factors are presented in Table 1. Compared to the cohort born 1901–02, the cohort born 1923–24 was slightly older, had more often more than compulsory education (i.e. 6 years), had higher mean MMSE score, was more often married, was more often living alone, had higher prevalence of stroke, and was less often living in sheltered housing.

**Table 1:** Characteristics of 85-year-olds examined in 1986–87 and 2008–10.

	Total		p
	1986–87	2008–10	
	% (N = 494)	% (N = 571)	
Age, mean (SD)	85.55 (0.12)	85.91 (0.17)	<0.001
Female sex	71.1 (351)	62.9 (359)	0.005
More than basic education, % (N)	25.2 (115/457)	57.7 (31/553)	<0.001
History of stroke, % (N)	18.8 (93)	27.1 (155)	<0.001
Mini Mental State Examination, mean (SD)	23.6 (7.7)	25.06 (6.45)	<0.001
Married, % (N)	23.9 (117/490)	35.1 (191/544)	<0.001
Sheltered living, % (N)	24.1 (119/494)	13.3 (76/571)	<0.001
Living alone, % (N)	57.1 (286/494)	66.8 (370/554)	0.003

SD = standard deviation.

### The prevalence of dementia and its subtypes

The prevalence of dementia was 29.8% in 1986–87 and 21.7% in 2008–10 (OR 0.66; 95%-CI: 0.50–0.86; Fisher’s exact test p = 0.003) (Table 2). The decline was similar when diagnoses were based on the neuropsychiatric examination alone or on the cut-off score <24 in the MMSE. The prevalence of mild dementia decreased from 8.3% in 1986–87 to 2.3% in 2008–10 (Fisher’s exact test p < 0.01). Mean age of onset was 79.5 years (SD = 5.08; median 81.00) in cohort 1901–02 and 80.1 years (SD = 4.5; median 81.00) in cohort 1923–24 (t-test; p = 0.321).

**Table 2:** The prevalence of dementia and its severity in 85-year-olds examined in 1986–87 and 2008–10.

	Dementia according to different methods								
	Dementia			Dementia (examination only)			MMSE below 24 points		
	1986–87% (N)	2008–10% (N)	OR (95%-CI)	1986–87% (N)	2008–10% (N)	OR (95%-CI)	1986–87% (N)	2008–10% (N)	OR (95%-CI)
Men	27.3 (39)	18.4 (39)	0.60 (0.36–0.997)	23.8 (34)	17.5 (37)	0.68 (0.40–1.14)	24.5 (35)	20.9 (43)	0.81 (0.49–1.35)
Women	30.8 (108)	23.7 (85)	0.70 (0.50–0.97)	24.5 (86)	19.5 (70)	0.75 (0.52–1.07)	31.0 (108)	22.6 (79)	0.65 (0.46–0.91)
All	29.8 (147)	21.7 (124)	0.66 (0.50–0.86)	24.3 (120)	18.7 (107)	0.72 (0.54–0.96)	29.1 (143)	22.0 (122)	0.69 (0.52–0.91)
	**Severity of dementia**								
	**Mild**			**Moderate**			**Severe**		
Men	7.7 (11)	2.4 (5)	0.29 (0.10–0.95)	9.1 (13)	8.5 (18)	0.93 (0.44–1.96)	10.5 (15)	7.5 (16)	0.70 (0.33–1.46)
Women	8.5 (30)	2.2 (8)	0.24 (0.11–0.54)	10.8 (38)	11.1 (40)	1.03 (0.65–1.65)	11.4 (40)	10.3 (37)	0.89 (0.56–1.43)
All	8.3 (41)	2.3 (13)	0.26 (0.14–0.49)	10.3 (51)	10.2 (58)	0.98 (0.66–1.46)	11.1 (55)	9.3 (53)	0.82 (0.55–1.22)

Note: Mini Mental State Examination (MMSE) was only done in 491 individuals in 1986–87.

The sample examined in 1986–87 is reference group for all odds ratio (OR).

Birth cohort changes regarding subtypes of dementia are given in Table 3. As may be seen, the decline in dementia prevalence was only significant for ‘vascular dementia only’ and vascular/mixed dementia in the total sample and among women (Table 3). However, all ORs for Alzheimer’s disease were in the same direction.

**Table 3:** The prevalence of types of dementia in 85-year- olds examined in 1986–87 and 2008–10.

	Alzheimer’s Disease incl mixed dementia			Alzheimer’s Disease only			Mixed dementia		
	1986–87% (N)	2008–10% (N)	OR (95%-CI)	1986–87% (N)	2008–10% (N)	OR (95%-CI)	1986–87% (N)	2008–10% (N)	OR (95%-CI)
Men	14.7 (21)	9.0 (19)	0.57 (0.30–1.11)	12.6 (18)	8.0 (17)	0.61 (0.30–1.22)	2.1 (3)	0.9 (2)	0.44 (0.07–2.69)
Women	19.4 (68)	16.7 (60)	0.84 (0.57–1.23)	15.1 (53)	13.6 (49)	0.89 (0.58–1.35)	4.3 (15)	3.1 (11)	0.71 (0.32–1.56)
All	18.0 (89)	13.8 (79)	0.73 (0.53–1.02)	14.4 (71)	11.6 (66)	0.78 (0.54–1.12)	3.6 (18)	2.3 (13)	0.62 (0.30–1.27)
	**Vascular dementia incl mixed dementia**			**Vascular dementia only**			**Other types of dementia**		
Men	7.7 (11)	7.5 (16)	0.98 (0.44–2.18)	5.6 (8)	6.6 (14)	1.19 (0.49–2.92)	7.0 (10)	2.8 (6)	0.39 (0.14–1.09)
Women	14.2 (50)	7.5 (27)	0.49 (0.30–0.80)	10.0 (35)	4.5 (16)	0.42 (0.23–0.78)	1.4 (5)	2.5 (9)	1.78 (0.59–5.36)
All	12.3 (61)	7.5 (43)	0.58 (0.38–0.87)	8.7 (43)	5.3 (30)	0.58 (0.36–0.94)	3.0 (15)	2.6 (15)	0.86 (0.42–1.78)

The sample examined in 1986–87 is reference group for all odds ratio (OR). Other types of dementia 1986–87: four alcoholic dementia, two Normal Pressure Hydrocephalus, one each with schizophrenia, severe physical illness, subdural hematoma, vitamin b12 defieciency, and five where the cause could not be determined.

Other types of dementia 2008–9: ten Parkinson’s disease, two Normal Pressure Hydrocephalus, one each with brain tumour, organic brain syndrome, and cause could not be determined.

### Stroke

As seen in Table 1, the prevalence of stroke increased between 1986–87 and 2008–10. However, as seen in Table 4, the odds for dementia in those with prevalent stroke decreased between the examinations. A logistic regression, including all individuals in the two birth cohorts, showed that the interaction term stroke*birth cohort (OR 0.42; 95%-CI 0.23–0.80; p = 0.008), prevalent stroke (OR 4.30; 95%-CI 2.69–6.90; p < 0.001), but not birth cohort (OR 0.78; 95%-CI 0.55–1.09; p = 0.140) or male sex (0.79; 95%-CI 0.58–1.08) were associated with dementia.

**Table 4:** Prevalence of dementia in relation to stroke in two birth cohorts of 85-year-olds.

	1986–87			2008–10			Interaction birth cohort*stroke for prevalence of dementia
	Prevalent stroke		OR (95%-CI)	Prevalent stroke		OR (95%-CI)	
	No	Yes		No	Yes		
	Prevalent dementia	Prevalent dementia		Prevalent dementia	Prevalent dementia		
	% (N)	% (N)		% (N)	% (N)		p
Men	23.5 (28)	45.8 (11)	2.8 (1.1–6.8)	16.2 (24)	23.4 (15)	1.6 (0.8–3.3)	0.31
Women	23.4 (66)	60.9 (42)	5.1 (2.9–8.9)	20.1 (54)	34.1 (31)	2.0 (1.2–3.5)	0.02
All	23.4 (94)	57.0 (53)	4.3 (2.7–6.9)	18.8 (78)	29.7 (46)	1.8 (1.2–2.8)	0.008

Odds ratio (OR) describes OR for dementia in prevalent stroke. No prevalent stroke is reference value for all odds ratio.

### The effect of education and stroke

We then examined the effect of education and stroke on the birth cohort differences in dementia prevalence (Table 5). In an unadjusted model (model 1), birth cohort was associated with lower odd ratio for dementia (OR 0.66; 95%-CI: 0.50–0.86).

**Table 5:** Logistic regression analyses regarding the association between the dependent variable dementia and the independent variables birth cohort, sex, education and stroke, and different interaction terms.

Independent variables	Model 1	Model 2	Model 3	Model 4
	OR (95%-CI)	OR (95%-CI)	OR (95%-CI)	OR (95%-CI)
Birth cohort	0.66 (0.50–0.86)	0.90 (0.66–1.22)	0.81 (0.59–1.11)	0.98 (0.68–1.41)
Male sex		0.81 (0.59–1.1)	0.79 (0.57–1.09)	0.80 (0.58–1.11)
More than basic education		0.70 (0.51–0.97)	0.69 (0.50–0.95)	0.70 (0.51–0.96)
Stroke			2.52 (1.82–3.49)	3.78 (2.28–6.29)
Stroke*birth cohort				0.50 (0.26–0.97)

Dementia is dependent variable in all analyses.

Interaction terms education*birth cohort (p = 0.63) and education*stroke (p = 0.97) were not significant and therefore removed from the models.

Another logistic regression (model 2), including sex, education (basic versus more than basic) and birth cohort showed that having more than basic education (OR 0.70; 95%-CI 0.51–0.97; p = 0.030), but not birth cohort (OR 0.90; 95%-CI 0.66–1.22; p = 0.481) or sex (0.81; 95%-CI 0.59–1.1; p = 0.185), were associated with the dependent variable dementia. There was no interaction education*birth cohort (p = 0.63), indicating that the effect of education was similar between cohorts.

Another model (model 3), including sex, education (basic versus more than basic), birth cohort and prevalent stroke, showed that having more than compulsory education (OR 0.69; 95%-CI 0.50–0.95; p = 0.025), and stroke (OR 2.52; 95%-CI 1.82–3.49; p < 0.001), but not birth cohort (OR 0.81; 95%-CI 0.59–1.11; p = 0.190) or sex (0.79; 95%-CI 0.57–1.09; p = 0.147), were associated with the dependent variable dementia. There was no interaction education*stroke (p = 0.99).

A final logistic regression (model 4), including sex, education, birth cohort, prevalent stroke, and the interaction prevalent stroke*birth cohort found that education (OR 0.70; 95%-CI 0.51–0.96; p = 0.029), prevalent stroke (OR 3.78; 95%-CI 2.28–6.29; p < 0.001), prevalent stroke*birth cohort (OR 0.50; 95%-CI 0.26–0.97; p = 0.040), but not birth cohort alone (OR 0.98; 95%-CI 0.68–1.41; p = 0.920), were related to the dependent variable dementia.

Marital status (OR 1.09; 95%-CI 0.76–1.57; p = 0.628) did not influence odds of dementia or the association between dementia and birth cohort in regression analyses including birth cohort, sex and marital status.

## Discussion

The prevalence of dementia declined substantially between 1986–87 and 2008–10 among 85-year-olds born 1901–02 and 1923–24 in Gothenburg, Sweden. The largest decline was observed for vascular dementia, despite an increased prevalence of stroke. The cohort effect almost disappeared after controlling for educational level and the interaction term stroke*cohort in the regression analysis, suggesting that the decline in dementia prevalence was largely attributable to the higher educational level (i.e. having more than basic education), and decreased odds ratio for dementia in the presence of stroke in the later-born cohort. The largest decline in prevalence was noticed for mild dementia. The influence of education, the decline in mild dementia, as well as the decreased association between stroke and dementia might be related to a larger cognitive reserve in later-born cohorts. The decreased association between stroke and dementia may also be attributable to better treatment of acute stroke. Our findings are similar to a recent incidence study from the Framingham Study^5^, in which the incidence of dementia, especially vascular dementia, declined, and the association between stroke and dementia decreased. However, the Framingham study could not assess the effect of educational level on the changing incidence of dementia due to the high educational level in its later born cohorts.

A decline in overall dementia prevalence from 1989–94 to 2008–2011 was also reported in the British MRC-FAS study^9^, in two US studies conducted 1982–1999^24^, and 1985–1994^25^, and a study from rural Sweden 1995–2003^26^. Two Swedish studies, conducted in septuagenarians between 1971–2005^27^ and in a population above age 75 between 1987–2004^28^, and a Spanish study in 70–84 year olds between 1989–96^29^ reported stable prevalence of dementia, while a Japanese studies conducted between 1985 and 2012^30, 31^, and a study from northern Sweden between 2000–02 and 2005–07^32^ reported increasing prevalence. Results from other parts of East Asia are inconclusive, maybe suggesting that later born birth cohorts have a higher prevalence^33, 34^ Regarding incidence, the Framingham Study^5^ recently reported a decline in the incidence of overall dementia and vascular dementia 1977–2013, the MRC Cognitive Function and Ageing Study (CFAS) a 20% decline in incidence of dementia mainly among men between 1989–94 and 2008–11^6^, and a French Study a decline in incidence of dementia in women between 1988–89 and 1999–2000^7^, while two other American studies conducted 1992–2001^35^, and 1997–2008^36^ found no change in the incidence of Alzheimer’s disease, and the Rotterdam study reported a non-significant decrease 1990–2000^37^. Reasons why some studies find that prevalence or incidence decline, while others report no changes or increasing prevalence, may be attributable to differences in how life-courses of birth cohorts are embedded in different historical contexts in different parts of the world^8^, or that the positive studies in general had a longer inter-cohort time and higher age of their samples. In a previous study on 70- and 75-year-olds born 1901–02/1906–07 and 1930^27^, we could not detect differences in the prevalence of dementia between cohorts. One reason could be that the prevalence of dementia in this age group is low and that we did not have statistical power to detect subtle changes. Another may be that dementia among younger old populations has a higher genetic loading than dementia in the oldest-old.

In contrast to the Framingham study^5^, we found that the decline in dementia prevalence was to a large extent explained by the higher proportion with more than basic education in the later born cohort, while the Framingham study only found a decreased incidence among persons with higher education. One reason may the very high educational level in Framingham, with a too low prevalence of low education in the later époques, which precluded analyses of the effect of educational level. As in Framingham, we found that the decline in dementia prevalence was mainly driven by a decreased prevalence of vascular/mixed dementia. In our study from 1986, the proportion of vascular dementia was higher than previously reported in western countries^23^. One reason for the decline may be better treatment of stroke and its risk factors. Despite this, the prevalence of stroke increased, as previously noted in 75 year-olds from 1976–2005^14^. One reason may be that while incidence of stroke declines, survival after stroke has increased dramatically^11^. The reason why vascular dementia prevalence declined was that the risk for dementia in relation to stroke decreased substantially, as also found in the Framingham Study^5^. Later born cohorts may have increased cognitive reserve due to e.g. better formal and informal education and better early nutrition, making them less vulnerable to consequences of brain disease^8, 9^. However, while education explained most of the birth cohort difference in dementia prevalence, it did not explain the decreased risk of dementia after stroke. Further support for the cognitive reserve hypothesis is that the proportion of mild dementia declined. According to this hypothesis, individuals with better cognitive reserve develop dementia later, but decline faster, when they develop dementia^19, 20^. Thus, the phases of preclinical and mild dementia may be shorter in later-born cohorts, as shown in two previous population studies^21, 22^.

The prevalence of Alzheimer’s disease did not change to the same extent as vascular dementia, despite that blood pressure, a risk factor for Alzheimer’s disease in the first cohort^38^, declined during the period^14^, and that other vascular risk factors were better treated in the later born cohort. One would have expected that cognitive reserve should have had the same protective effect on Alzheimer’s disease pathology as on cerebrovascular disease. In addition, it was recently shown, in an autopsy study from Switzerland, that brain amyloid deposition declined between 1972 and 2008, especially above age 85 years^39^. However, a Japanese study reported that both the clinical diagnosis of Alzheimer’s disease and the histopathology of neurofibrillary tangles, another hallmark of Alzheimer’s disease, increased from 1985 to 2012^30^.

Among the strengths of the study are the large samples of 85-year-olds examined two decades apart, the use of identical methods for examinations and diagnoses, including algorithmic diagnoses, and that the same researcher (IS) was leading both studies. There are also several possible limitations. First, although response rates were similar at both occasions, we cannot exclude the possibility that response rates among individuals with dementia differed between studies. Support for the latter is the fact that non-responders compared to responders in the later born birth cohort had lower three year survival rate (76.7% versus 83.4%) and higher hospital discharge diagnosis for depression (3.5% versus 1.5%), while other health parameters were similar. These differences between participants and non-participants in the later born cohort is not likely to explain the large differences in prevalence of dementia between the two birth cohorts. Second, although identical methods for examinations and diagnoses were used, including algorithms, a psychiatrist (IS) examined the first cohort, while psychiatric nurses examined the second. We cannot exclude the possibility that evaluations and interpretations changed over time. However, the first author, who did the examinations in 1986–87, trained the nurses and inter-rater reliability was high. Third, among those with dementia, 70% in the first cohort, but only 20% in the second had a CT scan. We therefore had to rediagnose the cohort born 1901–02 without using information from the CT-scan. We also reclassified cases who had hypoperfusion dementia in 1986–87, a diagnoses seldom used today. These changes resulted in a decline in the proportion of vascular dementia from 47% to 42% in the first cohort. Vascular dementia might therefore be underdiagnosed in persons with silent infarcts or white matter lesions, both related to dementia in the first cohort^40^. Although this bias should be similar in both cohorts, we cannot exclude the possibility that more infarcts were clinically silent in the second cohort due to increased cognitive reserve. Fourth, symptom criteria for vascular dementia were restricted to definite focal symptoms or signs, and we did not use other cerebrovascular pathologies, e.g. white-matter changes or severe cardiovascular diseases, in the diagnosis. This might have underestimated vascular dementia in both cohorts. Fifth, Alzheimer’s disease may be underestimated when both Alzheimer encephalopathy and vascular dementia contribute to dementia. We included mixed cases among the vascular dementias to point to a group where vascular factors probably contribute to the dementia syndrome^23^. Sixth, a smaller proportion of participants in the later-born birth cohort had a key informant interview (78% in 2008–10 versus 91% in 1986–87). This might have resulted in slightly lower prevalence in the later examination. Finally, the single-age group and the fact that the time trends were determined based on assessments at only two time points could be additional limitations of this study.

Most dementia cases occur after age 80. Our finding of a declining prevalence of vascular and mixed dementia might have important implications, as it shows, as suggested in the paper on the first birth cohort^23^, that this dementia is more amenable to prevention and treatment than Alzheimer’s disease. The decline was mainly explained by the higher proportion with more than basic education and the lower odds ratio for dementia in those with stroke in the later born birth cohort. Educational level increases worldwide. This may influence dementia prevalence by increasing cognitive reserve.

## Methods

### Samples

85-year-olds born 1901–02 and 1923–24 were examined in 1986–87 and 2008–10 (N = 1065). All samples were systematically obtained, based on birth dates, from the Swedish Population Register, which covers names and addresses of all people living in Sweden. The studies included persons living in private households and in institutions.

#### Cohort 1901–02

Every second 85-year-old in Gothenburg, Sweden, born July 1, 1901 to June 30, 1902 were invited to the examination in 1986–87 (n = 783)^23^. Forty-three individuals died before the examination, leaving an effective sample of 783, among which 494 (63.1%) (143 men and 351 women) participated. Non-participants and participants did not differ regarding sex, marital status, 3-year survival rate (71.3% versus 74.1%) and registration as psychiatric outpatient or inpatients in Gothenburg, as described previously^23^.

#### Cohort 1923–24

Every second 85-year-old in Gothenburg, Sweden, born July 1, 1923, to June 30, 1924, were invited to the examination in 2008–2010 (N = 1013). Forty individuals died before the examination, 19 could not speak Swedish, four had emigrated outside Sweden and six could not be traced, leaving an effective sample of 944 individuals, among which 571 (60.5%; 212 men and 359 women) participated. Non-participants and participants did not differ regarding sex (women 64.1% versus 62.9%), or hospital discharge diagnoses for cardiovascular disorders (35.9% versus 38.9%), stroke (8.3% versus 7.9%), and mental disorders (7.0% versus 4.2%). Non-participants had lower survival until age 88 years (76.7% versus 83.4%; p = 0.011), and higher prevalence of depression in the hospital discharge register (3.5% versus 1.2%; p = 0.022).

### Ethical approval and informed consent

Informed consent was obtained from all participants and/or their relatives. The Regional Ethical Review Board approved the study, and all methods were performed in accordance with the relevant guidelines and regulations.

## Methods

The examinations took place at an outpatient clinic or in the participant’s place of living, and included somatic, neuropsychiatric and neuropsychological examinations, key informant interviews, assessments of functional ability, sensory functions, social function, CT-scan of the head, and laboratory tests including ECG, and biochemical evaluations^23^.

The semi-structured neuropsychiatric examinations, performed by a psychiatrist in 1986–87 and by experienced psychiatric research nurses in 2008–10, included assessments of psychiatric symptoms, signs of dementia, tests of mental functioning (e.g. memory, proverbs, language, visuospatial and executive abilities, apraxia, construction, agnosia), the Mini Mental State Examination (MMSE)^41^ and the Alzheimer’s Disease Assessment Scale – ADAS^42^.

The first author (IS), who performed the examinations in 1986–87, trained and supervised the nurses. Inter-rater reliability for signs and symptoms used to diagnose dementia was tested by dual ratings by psychiatric research nurses or psychiatrists. Inter-rater agreement was 89.4–100.0% (kappa values 0.74–1.00).

Semi-structured interviews with close informants were performed in 451 participants (91%) in 1986–87 and in 443 (78%) in 2008–2010. The interviews comprised questions about changes in behavior and intellectual function (e.g. changes in personality, memory, intellectual ability, language, visuospatial function, psychiatric symptoms, activities of daily living), background factors (e.g. history of stroke/TIA, head trauma, alcohol abuse) and questions about onset age and course of dementia.

### Diagnostic procedures

The diagnostic procedures, described in detail previously^23^, were identical and done by the same psychiatrist (IS) both in 1986–87 and 2008–10. First, a diagnosis of dementia was made from the psychiatric examination and the close informant interview separately using an algorithm based on the DSM-III-R criteria^43^. Each symptom had to attain a level causing significant difficulties in social life. A final diagnosis was made from the combined information. Severity was registered according to DSM-III-R^43^.

Individuals with dementia were classified into etiological subgroups. Alzheimer’s disease was diagnosed according to NINCDS-ADRDA-criteria^44^. Vascular dementia was diagnosed similar to NINDS-AIREN-criteria^45^; i.e. when there was a temporal connection (within one year) between the first symptoms of dementia and a history of stroke/TIA. Mixed dementia was diagnosed when there was a history of stroke/TIA without clear temporal connection with dementia onset (more than one year). Other causes were diagnosed when dementia evolved in temporal connection with other disorders of sufficient degree to produce dementia.

Information on stroke/TIA was derived from self-reports, close informants and the Swedish hospital discharge register, as described previously^46^. Participant and key-informant interviews were structured, but allowed clarifying questions, and included questions about sudden onset of focal symptoms or acute aphasia, symptom duration, and age at stroke/TIA. Neuropsychiatrists evaluated information from the interviews, including side notes. Stroke/TIA was only diagnosed in cases with a definite history of acute focal symptoms (i.e. hemiparesis or aphasia). Information was also obtained from the Swedish Hospital Discharge Register, where all persons admitted to Swedish hospitals are registered according to the *International Statistical Classification of Diseases and Related Health Problems*. It has been shown that 94% of strokes in the Hospital Discharge Register are correctly classified^47^. Education was assessed from self-reports or close informants, and defined as 6 years mandatory or more.

### Statistical methods

Differences in proportions were tested with Fisher’s exact test or chi-square, and differences in means with t-test. The impact of different variables on dementia prevalence (dependent variable in all analyses) was tested with logistic regressions, including tests of interactions. The variables education, stroke and sex were chosen based on theoretical considerations as most likely to influence birth cohort differences in dementia prevalence. We first examined the effect of birth cohort on the prevalence of dementia, dementia severity and type of dementia stratified by sex and also analysed for the whole birth cohort (adjusted for sex). We also examined the effect of stroke on dementia prevalence stratified by birth cohort and sex, and tested the difference in OR between cohorts by analyzing the interaction stroke*birth cohort in the whole sample (also including stroke, birth cohort and sex as independent variables in the analyses), and also stratified by sex. Dementia was the dependent variable in these analyses.

Finally, we examined the effect of stroke, birth cohort, sex, and education on dementia prevalence in the whole sample using logistic regression models including interaction terms. Dementia was dependent variable in all these analyses. Model 1 was the unadjusted model. Model 2 included sex, education (basic versus more than basic), and birth cohort. Model 3 included sex, education (basic versus more than basic), birth cohort, and prevalent stroke. Model 4 included sex, education, birth cohort, prevalent stroke, and the interaction stroke*birth cohort. The interactions education*birth cohort and education*stroke were not significant and therefore removed from the models.

ORs were calculated and the two-tailed level of significance (p < 0.05) was used in all analyses, which were done with SPSS version 22.0 (IBM corp., Armonk, New York, USA).

### Data availability statement

The datasets generated during and/or analysed during the current study are available from the corresponding author on reasonable request.
